# Effect of Community-Based Kangaroo Mother Care Package on Neonatal Mortality Among Preterm and Low Birthweight Infants in Rural Pakistan: Protocol for a Cluster Randomized Controlled Trial

**DOI:** 10.2196/28156

**Published:** 2021-08-10

**Authors:** Shabina Ariff, Atif Habib, Zahid Memon, Tayyaba Arshad, Tariq Samejo, Ikram Maznani, Muhammad Umer, Amjad Hussain, Arjumand Rizvi, Imran Ahmed, Sajid Bashir Soofi, Zulfiqar A Bhutta

**Affiliations:** 1 Department of Paediatrics & Child Health Aga Khan University Karachi Pakistan; 2 Center of Excellence in Women & Child Health Aga Khan University Karachi Pakistan

**Keywords:** community kangaroo mother care, low birth weight, KMC champions, neonatal mortality, RCT protocol, Pakistan

## Abstract

**Background:**

Neonatal mortality due to preterm birth and low birthweight remains a significant challenge in Pakistan. Kangaroo mother care (KMC) is a unique, low-cost intervention proven to reduce neonatal mortality and morbidity and increase exclusive breastfeeding rates. However, KMC has not been attempted in community settings in Pakistan. We aim to implement and evaluate the effectiveness of a community-based KMC package to reduce neonatal morbidity and mortality among preterm and low birthweight (LBW) infants, which will provide evidence for policy development and the large-scale implementation of KMC across the country.

**Objective:**

The primary objective of this trial is to reduce neonatal mortality among preterm and LBW infants. The secondary objectives are growth (measured as weight gain), reduced incidence of possible serious bacterial infection, and increased exclusive breastfeeding and continued breastfeeding practices.

**Methods:**

We designed a community-based cluster randomized controlled trial in one rural district of Pakistan. Stable, LBW babies (weighing 1200 grams to 2500 grams) are included in the study. The community KMC package, consisting of the KMC kit, information and counseling material, and community mobilization through KMC champions (village volunteers), was designed after preliminary research in the same geographical location and implemented in intervention clusters. The standard essential newborn care is offered in the control clusters. Infants are recruited and followed up by independent teams of data collectors. Data are collected on the duration of skin-to-skin contact, growth, breastfeeding practices, morbidities, neonatal mortality, and neurodevelopment status. Data analysis will be conducted based on the intention to treat principle. The Cox regression model will be used to assess the primary outcome of neonatal mortality. The secondary outcomes will be evaluated using linear or logistic regression.

**Results:**

The Ethics Review Committee of Aga Khan University, Pakistan, approved the study protocol in February 2017. Data collection began in August 2019 and will be completed in December 2021. Data analyses are yet to be completed.

**Conclusions:**

This intervention may be effective in preventing sepsis and subsequently improve survival in LBW newborns in Pakistan and other low-income and middle-income countries worldwide.

**Trial Registration:**

clinicaltrials.gov NCT03545204; https://clinicaltrials.gov/ct2/show/NCT03545204

**International Registered Report Identifier (IRRID):**

DERR1-10.2196/28156

## Introduction

Neonatal mortality has emerged as a unique challenge for Pakistan. Although Pakistan has made progress in reducing infant and below 5 years mortality, little progress has been made to improve neonatal mortality in the last 3 decades [1-3]. The primary causes of neonatal mortality in Pakistan are birth asphyxia, sepsis, and preterm births [1,2]. Most neonatal deaths, especially those attributed to preterm births and low birthweight (LBW), can be averted by better coverage and low-cost, evidence-based interventions [4-7]. However, despite these interventions' availability and proven effectiveness, they have not been implemented on a large scale in Pakistan [8,9].

Kangaroo mother care (KMC) is a unique and low-cost intervention that significantly impacts preterm or LBW neonatal outcomes [10]. KMC was first initiated in 1978 by Dr. Edgar Rey in Bogotá, Colombia, who developed a technologically simple method defined as “early, continuous, and prolonged mother-infant skin-to-skin contact, with (ideally) exclusive breastfeeding.” UNICEF (The United Nations International Children’s Emergency Fund) reported this practice worldwide in 1983, and it was the first time the term “kangaroo’” was used to describe this practice [11]. In 2003, the WHO (World Health Organization) developed the first guidelines on the key aspects of KMC (kangaroo position, kangaroo feeding and nutrition strategy, and early discharge and strict ambulatory follow-up of KMC) [10].

Several studies have demonstrated the benefits of KMC in reducing neonatal morbidity and mortality and improving weight gain and exclusive breastfeeding rates [12-21]. Similarly, Lassi et al. [22] documented early initiation of breastfeeding, hygienic cord care, and KMC as effective neonatal infant and child mortality reduction interventions.

Despite high rates of home births in rural areas [23,24], KMC has never been tried in Pakistan’s community settings. The noncompliance to KMC practices can be best explained by various cultural factors inherent to religious and indigenous practices [25] in the community, including but are not limited to the covering of the body for modesty [23,26]. In addition, a low facility birth rate and a short post-delivery stay among rural communities are significant obstacles to initiating and sustaining KMC in the health facilities [27-29].

Given the high burden of neonatal deaths and the paucity of evidence on locally acceptable KMC, it must be tested in the community setting to generate the evidence to scale up its implementation across the country further. We propose to test the effectiveness of community KMC (cKMC) in our sociocultural context. A preliminary study was conducted to inform the design of the cKMC package and its implementation strategies. The strategies include delivering the KMC kit to mothers; garnering support for KMC; developing a buddy system to support mothers; establishing KMC champions (volunteers) within the communities; mobilizing communities using information, education, and communication (IEC) tools, including video messages and docudramas; and training community health workers on KMC and essential newborn care.

Based on these interventions, we aim to implement a cKMC package to reduce neonatal morbidity and mortality among premature and LBW infants. The primary objective of this trial study is to evaluate the effectiveness of cKMC in lowering neonatal mortality among premature and LBW infants. The secondary objectives include assessing the impact of cKMC on growth (measured as weight gain), the incidence of possible serious bacterial infection (PSBI) and referrals to the hospital, exclusive breastfeeding and continued breastfeeding practices, and neurodevelopmental assessments in a subset of recruited LBW babies at 6 and 12 months of age.

## Methods

### Study Design

**Figure 1:**
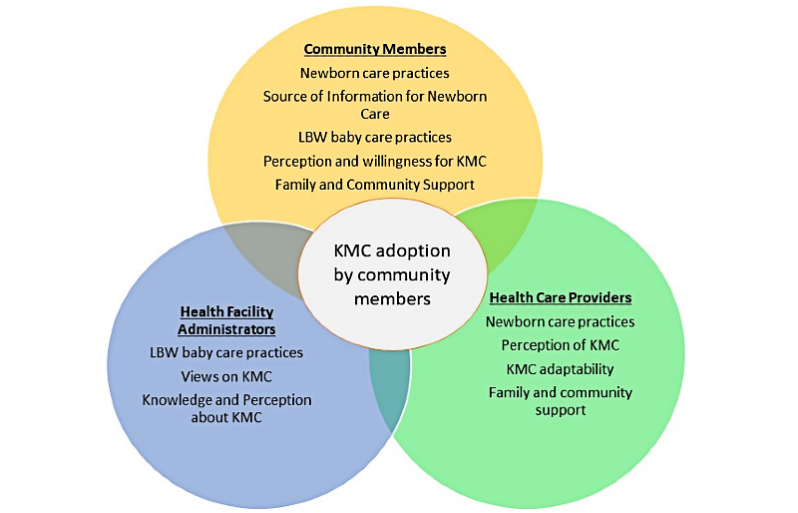
Conceptual framework and major themes from formative preliminary research. KMC- kangaroo mother care; LBW- low birth weight newborn.

We are conducting a cluster randomized controlled trial in one of the rural districts of Pakistan. The cKMC package has been developed based on preliminary research, involving in-depth interviews and focused group discussions with major stakeholders. A conceptual framework was developed based on the existing data to guide research themes (Figure 1).

### Study Site

The study is being conducted in 2 subdistricts (Taluka-Johi and Taluka-Khairpur Nathan Shah) of the district Dadu, which is a rural agrarian district in Sindh province of Pakistan. The overall population of the 2 talukas is about 2 million people residing in 54 union councils. The union council is the smallest administrative unit in Pakistan, with 15,000 to 25,000 people. The study area's population is largely poor, with 68% of households belonging to the lowest wealth quintiles. The study area also has an LBW prevalence of 27.7%, with an exclusive breastfeeding rate of 17.3%. Half of the women still deliver at home, and the proportion of facility births is 48.8% [30].

The public sector primarily provides the health care in the target area. There are 2 secondary care hospitals in the study area. There is a basic health unit (BHU) in each union council, and 15 to 20 lady health workers (LHW) are affiliated with each BHU, serving as frontline health care providers for a population of 1000 people in their respective areas.

### Study Population

#### Inclusion and Exclusion Criteria

All stable LBW newborns weighing 1200 grams to 2500 grams are screened within a 72-hour window, followed by enrollment after informed consent to participate in the trial is obtained. Newborns tolerating oral feeding with no respiratory distress, the absence of any symptoms of disease, and the absence of congenital anomalies are included in the study.

Whereas newborns weighing less than 1200 grams and with symptoms of disease according to predefined criteria (ie, unable to tolerate oral feeding; severe respiratory distress, including respiratory rates of less than 20 breaths per minute or more than 60 breaths per minute; grunting–central cyanosis; severe chest in-drawing; convulsions; unconsciousness; severe hypothermia of less than 32°C; apnea; and congenital malformation) are excluded and referred to the nearest health facility for management.

#### Sample Size

We considered union councils in the talukas as the clusters for our trial. The union council comprised a population of 25,000, with expected 29 births per 1000 people. We anticipated 200 LBW births per cluster, given the 27.7% prevalence of LBW in the study area [30]. Literature suggests 13.3% of the LBW infants die in the neonatal stage [31]. With an expected 30% reduction in mortality, 12 clusters (union councils) were needed per arm (a total of 24 clusters for the trial) to achieve 90% power and a 5% significance level. We estimated 200 births with a birth weight of less than 2.5 kg per cluster. A total of 4800 participants in the intervention and control groups are required to complete the study.

#### Randomization

**Figure 2:**
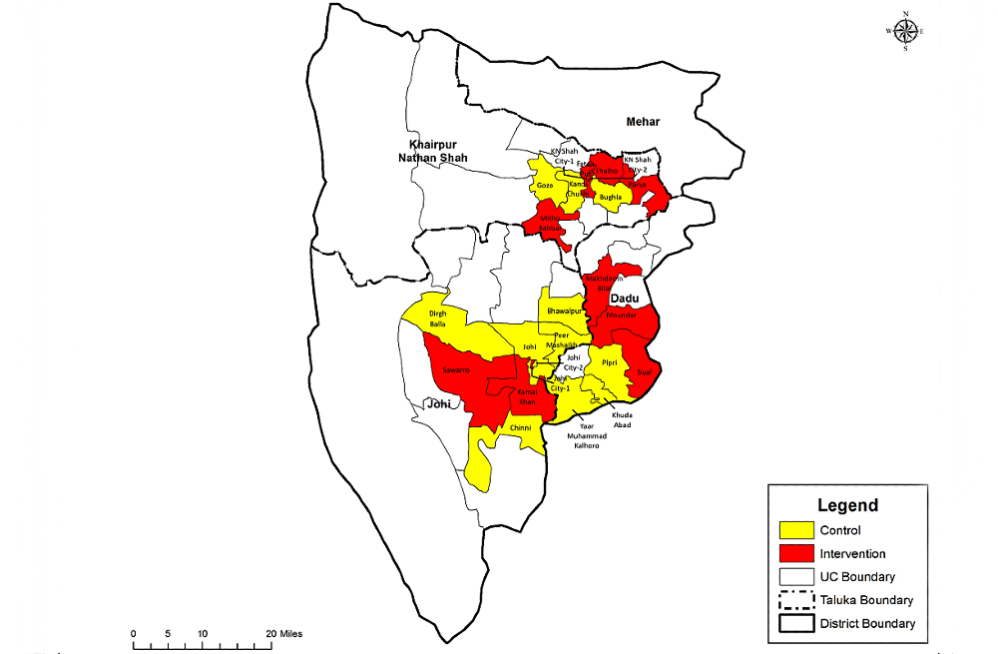
Study site and selected clusters. UC: union councils.

The 2 targeted talukas have a total of 54 union councils. Out of these, 24 were randomly selected by an independent researcher using a computer-generated program (Figure 2).

The clusters (union councils) were randomized using a restricted randomization scheme using the following indicators: population, live birth, the prevalence of LBW, neonatal mortality, skin-to-skin contact, breastfeeding practices, and distance from the taluka hospital. We conducted a baseline survey of the study area to collect data on these indicators. Blinding is not possible because of the nature of the intervention; however, to minimize measurement bias on the effect of the intervention, the data collection team is independent of the implementation team.

### Procedures

#### Pregnancy Surveillance and Birth Notification

Pregnancy surveillance was instituted as a continuous activity in the trial to identify and track new and existing pregnancies. A team comprising of 2 female community health workers per union councils is responsible for surveillance and birth notifications. Identified pregnancies in the intervention clusters are counseled on the KMC intervention and its benefits to mother and baby. At the same time, counseling on essential newborn care is given in the control areas.

The team also records pregnancy outcomes (ie, miscarriage, stillbirths, and live births), and all registered live births are followed up for mortality outcomes at 28 days of life. Other additional sources for birth notifications are the female health workers, village elders, and traditional birth attendants, who support the study teams and provide regular reports on births in their respective areas.

#### Screening, Recruitment, and Intervention Delivery

When a birth is reported, the recruitment and intervention teams will visit the household within 72 hours of the delivery. The screening and recruitment are carried out by a separate team, comprising of a male team leader and 2 community health workers. Once the eligibility criteria are fulfilled and consent from the mother or caregiver is recorded, the mother-baby dyad is recruited in the study. After recruitment, the team visits the household on days 5, 7, 10, 14, 21, and 28 in the intervention clusters to support KMC practice. They will demonstrate the steps of KMC, including KMC positioning with the support of a “chaddar” (a cloth that females use to cover their heads and body for modesty) and IEC material. The LBW babies in the control clusters will receive standard essential neonatal care as per the national guidelines. The intervention team also conducts 1-on-1 and community-based mobilization for KMC advocacy.

#### Data Collection

Independent data collection teams comprising of 2 community health workers in each cluster will be deployed in both intervention and control clusters. The teams collect data on KMC compliance, anthropometry (weight and length), signs of PBSI, breastfeeding practices, and mortality on scheduled follow-ups at days 7, 28, and 59. On days 120 and 365, information on mortality, breastfeeding practices, infant and young child feeding practices, and nutrition status (weight and length) will be captured. In addition, a neurodevelopmental assessment will be performed on a subset of children using the Bayley’s scale at 12 months [32].

The data collection teams examine the baby for any symptoms of disease during each household visit. If symptoms of disease are observed, a prompt referral will be made to the nearest health facility. Participant mothers willing to comply but unable to perform KMC for 1 week or more due to illness or other reasons are excluded from the study. Loss to follow-up is defined as the unavailability of a mother-baby dyad for 3 consecutive follow-ups after recruitment.

#### KMC Intervention Package

A cKMC package is developed to support mothers and overcome sociocultural barriers to practicing KMC. The package includes the following:

##### KMC Kit

The kit contains 20 diapers for the child, 10 napkins for the mother, 1 towel, a pair of socks and cap for the infant, 1 bar of soap, and an educational brochure in the local language. These items are packaged in a ziplock plastic bag. The recruitment team is responsible for providing the kit to enrolled mothers. 

##### Education Package

We have developed a contextual IEC package for parents and families to create awareness and describe the benefits of KMC for the survival and well-being of LBW newborns. The material comprises flip charts, wall mounts, and a self-explanatory video on the steps of KMC, its benefits, and the potential implementation of a buddy system (ie, skin-to-skin contact provided by other family members).

##### Community Mobilization (KMC Champions)

A community mobilization team (1 male and 1 female) conducts one-on-one and group sessions concerning essential newborn care and KMC practices with newly pregnant women, mothers, and mothers-in-law. The sessions are conducted at regular intervals. The male mobilizer is responsible for the one-on-one and group sessions advocating KMC with fathers and other male members of the community.

The community mobilization team also encourages the recruitment of volunteers to function as KMC champions. The local community members serving as KMC champions serve as catalysts for mobilization. The mobilization staff also identifies and recruits cochampions (other community volunteers) to be mentored by KMC champions. This group of local community members serve to disseminate KMC practices and facilitate uptake in the community.

A simple color-coded KMC calendar depicting 24 hours was designed in the local language for families of enrolled newborns to record the number of hours that the mother or buddy practices KMC. The mother and family members are instructed on how to use the calendar and asked to mark the number of hours KMC is practiced each day on the calendar. These data are collected at the end of each week.

### Outcome Ascertainment

The data are collected in a structured electronic format to ascertain outcomes. The anthropometric measurements are done per standard anthropometry guidelines [33] by the pair of measurers (weight and length). Infant weight is measured on pan scales (model 354; Seca) and length is measured by the infantometer (model 417; Seca). The details of the outcome measures are described in Textbox 1.

**Outcome measures and definitions. LBW: low birthweight; PSBI: possible serious bacterial infection; EBF: exclusive breastfeeding defined as the percentage of infants aged 0 to 6 months who are exclusively breastfed; KMC: kangaroo mother care.**

- **Reduction in neonatal mortality:** the reduction of mortality in LBW newborns during the first 28 days of life.
- **Improvement in growth (nutrition status):** the increase in newborn weight gain from birth and at days 14, 28, 59, 120, 180, and 365; and the increase in the length of the newborn from birth and at days 180 and 365.
- **Reduction in PSBI incidence:** the reduction in PSBI incidence during the neonatal period (days 14 and 28) and 59 days of life.
- **Improved EBF:** increase in the EBF rate up to 50% at 6 months of age.
- **Improved neurodevelopment:** KMC improves neurodevelopment outcomes while impairments in physical growth and brain and central nervous system development can result in cognitive, language, motor, neurosensory impairments, and behavioral disorders. Hence assessment will be done at 12 months of age.

### Training of Study Teams

The study investigators provided extensive training to the field teams regarding their assigned tasks. All staff received training on good clinical practice, and the pregnancy surveillance and birth notification teams received training on survey procedures and appropriate documentation. The implementation team received comprehensive training on the KMC package, implementation, and counseling; they were also trained on screening and recruitment procedures and referral protocols.

The data collection team was trained on interviewing techniques and data documentation using a handheld device. The training also included newborn examinations, recognizing symptoms of disease, prompt referrals, ascertaining KMC compliance, breastfeeding practices, and anthropometric measurements using the standard methodology and standardization processes [33]. The team was also trained to calibrate anthropometric instruments regularly using the standard measurement rods and weights.

The LHWs are the frontline health workers in the public sector employed by the Ministry of Health. The LHWs in the intervention clusters received orientation on the KMC intervention and standard essential newborn care. In contrast, LHWs in the control clusters were trained on standard essential newborn care only.

### Data Management

A data collection application was developed to collect the data on recruitment and outcome measures during follow-ups. These applications have a built-in range and consistency checks. If there are specific queries, the data is returned to the respective teams, and the query is resolved within 48 hours of data collection. The data are transferred to the Aga Khan University (AKU) secure data servers at the data management unit daily. A trial flow was developed, detailing the number of participants through assessments of eligibility, randomization, follow-up, and analysis. Reasons for exclusions and withdrawals are appropriately explained and documented.

### Data Analysis

For data analysis, we will use the intention to treat approach using STATA software (version 17; StataCorp). Data will first be analyzed using person-time as the denominator for the primary outcome (neonatal mortality between enrollment and 28 days of age). Hazard ratios and 95% CIs will then be calculated using a Cox regression model to evaluate the effect of the intervention (cKMC) on infant deaths. We will also estimate the impact of cKMC using the number of enrolled infants as the denominator to deduce risk ratios using generalized linear models of the binomial family with a log link function. The summary data for background characteristics in the intervention and control groups will be presented as means and proportions.

The effect of KMC on secondary outcomes (ie, exclusive breastfeeding, weight and length gain, the incidence of illnesses and hospitalizations, and care-seeking behavior) will be assessed using linear or logistic regression after adjusting for clustering in the case of twins or another enrolled baby subsequently born to the same mother, as well as other potential confounders.

### Monitoring and Evaluation

The study investigators and technical staff from the AKU will interact with the study team through regular field site visits to review the study process and progress. The study managers will share weekly reports. All key areas will be monitored, including the enrollment rate, timing of the intervention delivery initiation, consent procedures, referrals and follow-up visits, and timely transmission of data to AKU.

## Results

The Ethics Review Committee of Aga Khan University, Pakistan, approved the study protocol on February 15, 2017 (ID. 4467-Ped-ERC-16). In addition, ethical clearance was sought from the National Bioethics Committee, Pakistan. The trial is registered with clinicaltrials.gov: NCT03545204. Data collection began in August 2019 and will be completed in December 2021. Data analyses are yet to be completed. The datasets used for the article and the study is available from the corresponding author on request.

## Discussion

Despite the robust evidence supporting the use of KMC for preterm and LBW survival, scaling-up of KMC has proven an elusive goal for Pakistan and other low-income and middle-income countries for the last 40 years [34]. However, with increased awareness concerning the magnitude of newborn mortality among preterm and LBW infants, our trial anticipates providing evidence on the impact of initiating cKMC in the remote areas of Pakistan, where incubator care is inaccessible. Moreover, the benefits of performing KMC in the community setting will also be emphasized, facilitating the much-needed uptake of this intervention within rural communities.

Most of the evidence that favors KMC is derived from hospital-based settings; however, a recent study concluded that cKMC substantially improves neonatal and infant survival in low-income countries. KMC in community settings for infants with LBW could substantially reduce neonatal and infant mortality [18]. Furthermore, research carried out in Haryana, India, proposed cKMC was feasible and acceptable, with high adoption rates observed in mothers of LBW babies [35]. Similarly, a study conducted in Pakistan demonstrated that a package of interventions that included essential newborn care, chlorhexidine, and KMC reduced the risk of neonatal infection and omphalitis and positively impacted weight gain [19]. Although there is some evidence in favor of cKMC in low-income countries, it is imperative to conduct robust research on the impact of cKMC in Pakistan for its large-scale implementation.

There is a need to adopt community-based KMC in Pakistan’s rural areas, where most deliveries occur at home [3]. Our preliminary research showed a high acceptance rate of KMC in a community setting, with a willingness to perform KMC for at least 8 hours at home with family support. However, community mobilization was critical to resolve barriers and to achieve acceptance rates within the community. We are also focusing on pregnancy surveillance through which pregnant women are identified via door-to-door surveillance, and newborns are identified by an early birth notification system and follow-up at home. In addition, well-trained community health workers such as KMC champions carry out regular sessions in the community to develop mother and father champions and sensitize the community. Besides KMC champions, we intend to see the effectiveness of community KMC on neonatal mortality in LBW babies by engaging the LHW program through this study. The LHW program in rural Pakistan is the backbone of primary health care, including maternal and child health, and covers approximately 60% of the rural population [36].

Although there is considerable evidence on the effectiveness of KMC, previous trials were conducted in a controlled environment, where the results cannot be generalized to programs operating under field conditions. The objective of our trial is to scale up KMC practice in the remote areas of Pakistan and test this model, which can then be delivered by the health care providers employed in the public sector such as LHWs, lady health supervisors, community midwives, and lady health visitors. The findings of this study will provide enough evidence to develop policies and programs aimed at preventing neonatal mortality and improving maternal and child health and growth outcomes in poor resource settings.

cKMC intervention may be effective in preventing sepsis and subsequently improve survival in LBW newborns in Pakistan and other low-income and middle-income countries worldwide.

## Abbreviations

AKU: Aga Khan University
BHU: basic health unit
cKMC: community kangaroo mother care
IEC: information, education, and communication
KMC: kangaroo mother care
LBW: low birthweight
PSBI: possible serious bacterial infection
LHW: lady health worker
