# Correction: Effect of Community-Based Kangaroo Mother Care Package on Neonatal Mortality Among Preterm and Low Birthweight Infants in Rural Pakistan: Protocol for a Cluster Randomized Controlled Trial

**DOI:** 10.2196/104854

**Published:** 2026-07-02

**Authors:** JMIR Publications Editorial Office

**Affiliations:** 1 JMIR Publications Toronto, ON

Following publication of “Effect of Community-Based Kangaroo Mother Care Package on Neonatal Mortality Among Preterm and Low Birthweight Infants in Rural Pakistan: Protocol for a Cluster Randomized Controlled Trial” [1], concerns were raised regarding a potential conflict of interest between reviewer I Hussain and members of the author list. Following review, it was determined that the authors and the reviewer were both affiliated with Aga Khan University. This conflict of interest was not disclosed to the editorial office as per journal policy at submission.

This article has been subsequently re-assessed by a *JMIR Research Protocols* editor who determined that the content of this review did not unduly influence the decision to accept the article. The decision to accept the submission was based primarily on the remaining reviewers’ comments and the previous editor’s contemporary appraisal of the manuscript.

As such, the JMIR Publications Editorial Office issues this corrigendum to update the Conflicts of Interest statement:

Reviewers were found to have undisclosed conflicts of interest with members of the author list under the terms of JMIR Publications policy. The editor in chief determined that the content of the reviews did not unduly influence the decision to accept the article.

We regret that these issues were not identified before publication.

The correction will appear in the online version of the paper on the JMIR Publications website together with the publication of this correction notice. Because this was made after submission to PubMed, PubMed Central, and other full-text repositories, the corrected article has also been resubmitted to those repositories.
